# The Relationship among Oceanography, Prey Fields, and Beaked Whale Foraging Habitat in the Tongue of the Ocean

**DOI:** 10.1371/journal.pone.0019269

**Published:** 2011-04-27

**Authors:** Elliott L. Hazen, Douglas P. Nowacek, Louis St. Laurent, Patrick N. Halpin, David J. Moretti

**Affiliations:** 1 Duke University Marine Lab, Beaufort, North Carolina, United States of America; 2 Joint Institute for Marine and Atmospheric Research, University of Hawaii, Honolulu, Hawaii, United States of America; 3 Pacific Fisheries Environmental Lab, National Oceanic and Atmospheric Association, Pacific Grove, California, United States of America; 4 Pratt School of Engineering, Duke University, Durham, North Carolina, United States of America; 5 Woods Hole Oceanographic Institution, Woods Hole, Massachusetts, United States of America; 6 Naval Undersea Warfare Center, Newport, Rhode Island, United States of America; Institute of Marine Research, Norway

## Abstract

Beaked whales, specifically Blainville's (*Mesoplodon densirostris*) and Cuvier's (*Ziphius cavirostris*), are known to feed in the Tongue of the Ocean, Bahamas. These whales can be reliably detected and often localized within the Atlantic Undersea Test and Evaluation Center (AUTEC) acoustic sensor system. The AUTEC range is a regularly spaced bottom mounted hydrophone array covering >350 nm^2^ providing a valuable network to record anthropogenic noise and marine mammal vocalizations. Assessments of the potential risks of noise exposure to beaked whales have historically occurred in the absence of information about the physical and biological environments in which these animals are distributed. In the fall of 2008, we used a downward looking 38 kHz SIMRAD EK60 echosounder to measure prey scattering layers concurrent with fine scale turbulence measurements from an autonomous turbulence profiler. Using an 8 km, 4-leaf clover sampling pattern, we completed a total of 7.5 repeat surveys with concurrently measured physical and biological oceanographic parameters, so as to examine the spatiotemporal scales and relationships among turbulence levels, biological scattering layers, and beaked whale foraging activity. We found a strong correlation among increased prey density and ocean vertical structure relative to increased click densities. Understanding the habitats of these whales and their utilization patterns will improve future models of beaked whale habitat as well as allowing more comprehensive assessments of exposure risk to anthropogenic sound.

## Introduction

Beaked whales are one of the least understood marine mammal taxa in the world's oceans, particularly the *Mesoplodon* genera, all of which are listed as data deficient under the IUCN red list [1]. Due to their incredibly long and deep dives (e.g. > 50 minutes and 1000 meters), they have been extraordinarily difficult to study using typical visual techniques [2], [3]. Moreover, while *Mesoplodon* species are found in most of world's oceans, they are often distributed offshore necessitating the use of large ships to study their behavior and distribution [3]. Testament to the difficulty in studying them, ecological studies have been able to focus on little more than data from stranded animals, e.g. diet from stomach contents [4]. Beaked whale species also appear to be particularly sensitive to mid-frequency sonar as a number of mass strandings have occurred coincident with naval exercises [5]. The lack of information on beaked whale ecology has made assessing the potential risk from anthropogenic activity much more difficult. Recent research using short-duration tags around oceanic islands (i.e., where deep waters are close to shore) has provided valuable and insightful data on diving behavior, beaked whale echolocation, and identifying beaked whale prey [6], [7], [8].

Vessel surveys along the western side of Abaco Island, Bahamas and in the Tongue of the Ocean have established what may be a resident population of Blainville's beaked whales inhabits these waters [3], [9]. The Tongue of the Ocean (TOTO) is a deep-water basin approximately 204 kilometers long and 36 kilometers wide and varying in depth from 1280–2010 meters. The semi-enclosed nature of the TOTO makes it an ideal study site as it contains bathymetric features that include known habitat for Blainville's beaked whales. The TOTO is especially conducive for studying beaked whales because it is home to a large bottom-mounted hydrophone array that is part of the Atlantic Undersea Test and Evaluation Center (AUTEC). Moored hydrophones have been commonly used to understand patterns of vocal animals across a range of temporal scales (e.g. daily, monthly, seasonal [10]). Blainville's beaked whales have very regular and predictable inter-click intervals making species identification possible from an acoustic recording [2], [11]. Foraging click trains have been detected successfully using a combination of manual and automated methods from moored hydrophones in the TOTO [12], [13].

Deep scattering layers (DSLs) serve as an important prey resource for top predators, particularly in oligotrophic oceanic habitats [14], [15], [16] and their vertical migration may serve as an important source of mixing in the ocean [17]. The composition of species in deep scattering layers is diverse and can change both temporally and spatially requiring multiple samples and sampling gear [18], [19], [20], [21]. The functional groups comprising DSLs include myctophid fishes and squid, both of which are known to be prey items of Blainville's beaked whales [4], [8]. Traditional net tows give limited information on depth distributions and average biomass throughout the length of a trawl but allow measurements and species identification of sampled organisms. In contrast, fisheries acoustics offer a minimally invasive approach to measure sound scattering organisms and to provide an acoustic density of prey. When possible, trawl samples can be used to ground truth the acoustically detected organisms including length frequency distributions and species compositions [22]. Acoustic data in the absence of trawls can still provide a relative measure of prey distribution [19], [23] that may help inform models of top predator distribution.

Habitat models are a valuable tool that can help identify factors structuring organismal distribution, abundance, and even behavior [24]. In addition, habitat models are a valuable tool for identifying critical habitat and assembling spatial management strategies for highly mobile species [25]. Habitat models for beaked whales have been created in the Bahamas, eastern tropical Pacific, and Hawaiian islands though most have focused on depth as the primary factor in determining preferable habitat [3], [9]. Some recent models have used oceanographic parameters from *in situ* sampling [26], [27]. Previous researchers have modeled marine mammal acoustic recordings as a function of environmental correlates for rorqual whales and dolphins however most of these recordings document social behaviors rather than foraging events [28], [29], [30]. A multi-directional bottom-mounted hydrophone was deployed at Cross seamount southwest of the Kona coast and found a high number of beaked whale foraging clicks that were coincident with increased prey density [31].

The primary goals of this research were to (1) obtain fine scale spatial and temporal measurements of both prey and beaked whale foraging effort, (2) analyze spatial and temporal scale dependence in patterns of the deep scattering layer, (3) incorporate prey and oceanographic data in models of *Mesoplodon* habitat in the TOTO. By combining measurements of prey, acoustic recordings of *Mesoplodon* foraging behavior, and physical oceanography, we were able to examine trophic interactions and identify habitat characteristics that can be used to further assess and protect this deep diving and poorly understood species.

## Methods

**Figure 1:**
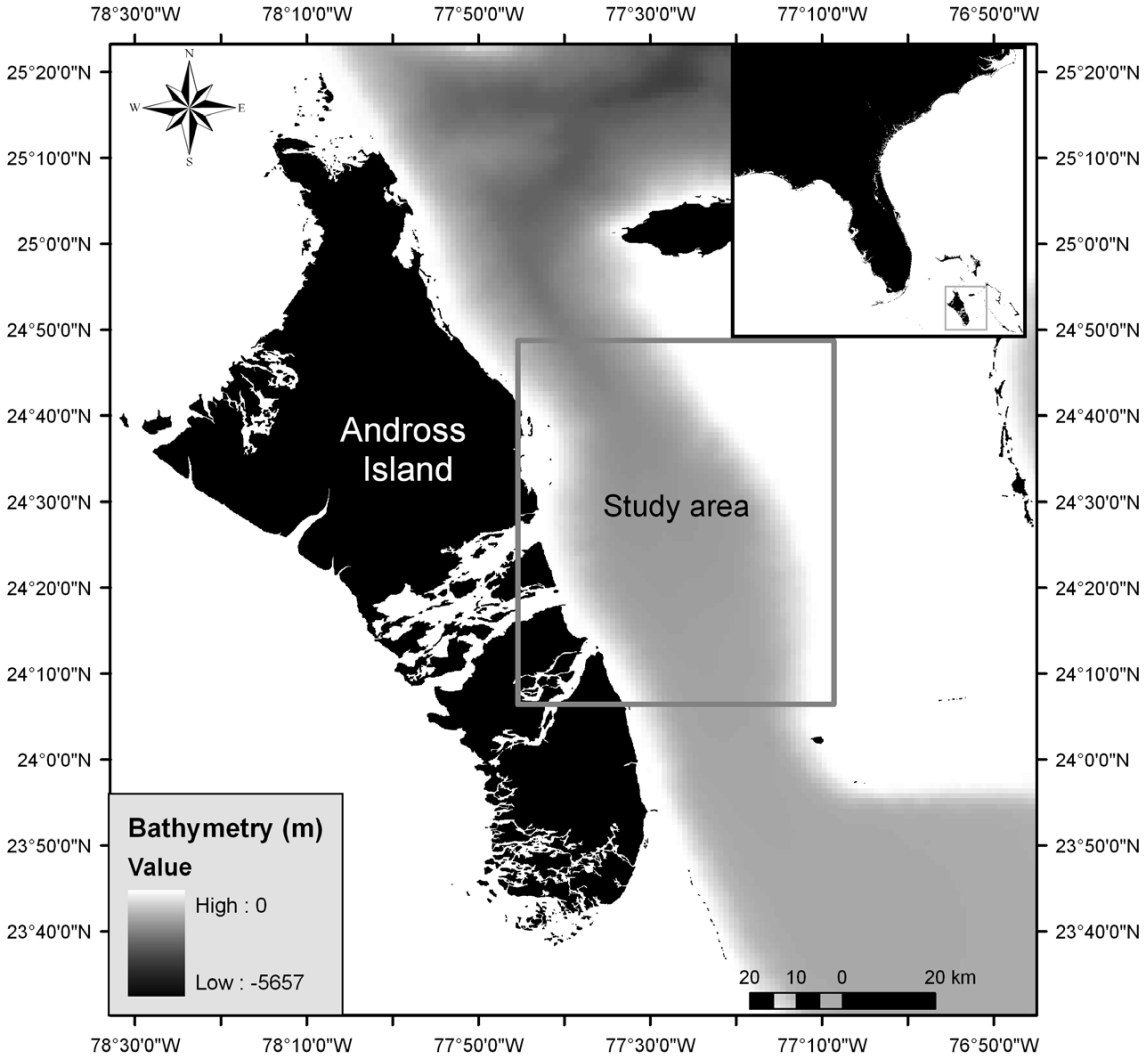
The Tongue of the Ocean and study site off the eastern coast of Andross Island, in the Bahamas.

We conducted fisheries acoustic and hydrographic surveys aboard the *R/V Revelle* (84 m beam length) to examine the distribution of the DSL in the TOTO, Bahamas from September 12^th^–October 2^nd^, 2008. The surveys consisted of three east-west cross-basin transects, two N-S along-basin transects, and 8 cloverleaf patterns (Figure 1). Each clover pattern consisted four 5 km transects to examine scale dependence and isotropy along each transect as well as temporal patterns at the intersection of each clover. Each clover was centered on or near bottom-mounted hydrophones to correlate with beaked whale detections.

Acoustic data (38 kHz) were primarily collected at night with one of the clovers and two of the transects surveyed during the day. The SIMRAD EK60 38-DD echosounder (7° beam width) was mounted in the transducer tube and was calibrated before the cruise using standard calibration procedures [32]. All acoustic data were collected using 2 kW transmit power with a 2048 s pulse width, and were processed in 500 m×10 m bins for exploratory analysis. Based on geostatistical techniques described below, all acoustic data were re-binned in 1 km×200 m grid cells for model input. Data were processed using a −90 dB threshold from the beginning of the far field until noise levels became apparent (5 to 1000 m). The data were manually scrutinized for noise spikes or regions of double echoes with bad data values excluded from analysis. Bottom echoes were excluded using an 8 ping filter to smooth the detected bottom with a backstep of 0.5 meters above the detected bottom when shallower than 1000 m. Scattering layer density was summarized using Sv a logarithmic relative density of acoustic scattering organisms (dB) and converted into NASC a linear measure of acoustic density (m^2^·nmi^−2^) for arithmetic operations and analyses. In addition, a single target detection algorithm with a −70 dB threshold was used to detect individual scattering organisms in the water column [33]. We were unable to tow nets to sample species distributions of the scattering layer but instead used the acoustic backscatter intensity to represent relative density of potential prey.

Physical data were collected using an autonomous deep microstructure profiler (DMP) for turbulence measurements, a conductivity-temperature-depth sensor (CTD), and expendable bathy-thermographs (XBTs). The CTD had salinity and temperature probes on board and was lowered up to 1000 meters in depth or within 50 meters of the bottom. The DMP was released and retrieved three times per clover and per transect to measure turbulence, diffusivity and water column environmental data with depth. Sensors on the profiler included a standard CTD, as well as dual airfoil probes to measure microstructure shear. These data were used to calculate turbulence dissipation rates and diffusivity in the water column (cm^2^·s^−1^, e.g. St. Laurent and Thurnherr 2007). We conducted 13 total CTD and XBT casts in addition to 36 DMP casts providing good spatial coverage of measured temperature and salinity data with depth over our study area.

Acoustic data from the 82 bottom mounted hydrophones of the AUTEC range were simultaneously recorded digitally at a 96 kHz sampling rate. Hydrophones were approximate 1 nautical mile apart except for two finer scale 7-hydrophone arrays. A multi-stage FFT based energy detector has been successfully used for detection of clicks from a variety of echolocating odontocetes including Blainville's beaked whales [12], [34]. We used an automatic click detector with an inter-click interval criterion of (0.15–1 s) to quantify Blainville's beaked whale clicks, which were subsequently manually scrutinized [11], [13]. The total hours of detected foraging click trains recorded during our survey were summed for each hydrophone to get a relative measure of foraging effort, which became the response variable for our habitat models. Previous research [6], [8], [11] has provided solid evidence of the links between foraging effort and echolocation, including ‘regular’ clicks and prey capture or ‘feeding buzzes’. These results allowed us to reliably assign acoustic data to foraging vs. non-foraging behavior for whales on AUTEC.

Spatial associations between foraging click rates, oceanography, and prey data were performed using ArcGIS 9.3 [35]. Although data were collected north of TOTO and towards Abaco bank, the hydrophone coverage restricted analysis to waters east of Andros Island and west of the Nassau bank in the Bahamas. All data were projected in Universal Transverse Mercator zone 18 and analyzed in 1 km×1 km horizontal grid cells. This resolution was chosen to minimize data gaps between hydrophones in the TOTO while ensuring adequate resolution for fisheries acoustic and oceanographic data. Bathymetric data were obtained from the Naval Undersea Warfare Center's hydrophone locations as the acoustic data were processed at each of these features. Semivariograms of prey density were used to identify the key spatial scales horizontally and vertically as an input for the geostatistical analysis [36]. To account for autocorrelation in our predictor variables and create uniform surfaces, each dataset was interpolated using a universal kriging function [37], [38]. This approach allowed us to visually identify hotspots and coldspots in each variable. Each hydrophone location was used to sample the acoustic surface, bottom depth, and microstructure diffusivity as inputs for the models of click density as a function of prey and environment.

Models of foraging intensity as a function of prey metrics, as a function of environmental variables, and as a function of all variables were fitted in R 2.10 [39]. Our response variable was total duration of clicking at each hydrophone concomitant with our cruise, and predictor variables included bottom depth (m), salinity, temperature (°C), diffusivity (cm^2^/s), backscattering density (dB), single target size (dB), and number of single targets in 1 km×1 km×200 m deep bins. Three model forms were fit to examine factors influencing acoustically measured foraging effort:

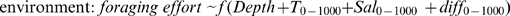
;

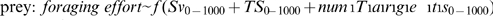
;

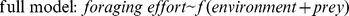
;

We used an iterative regression technique starting with a full model and selectively removing individual terms for both generalized linear (GLM) and generalized additive models (GAM) to fit the data and found the relationships to be largely linear with no additional deviance explained from the additive models. The best performing generalized model was chosen as a function of Akaike's Information Criterion (AIC, [40]) value for prey models, environmental models, and combined models.

## Results

**Figure 2:**
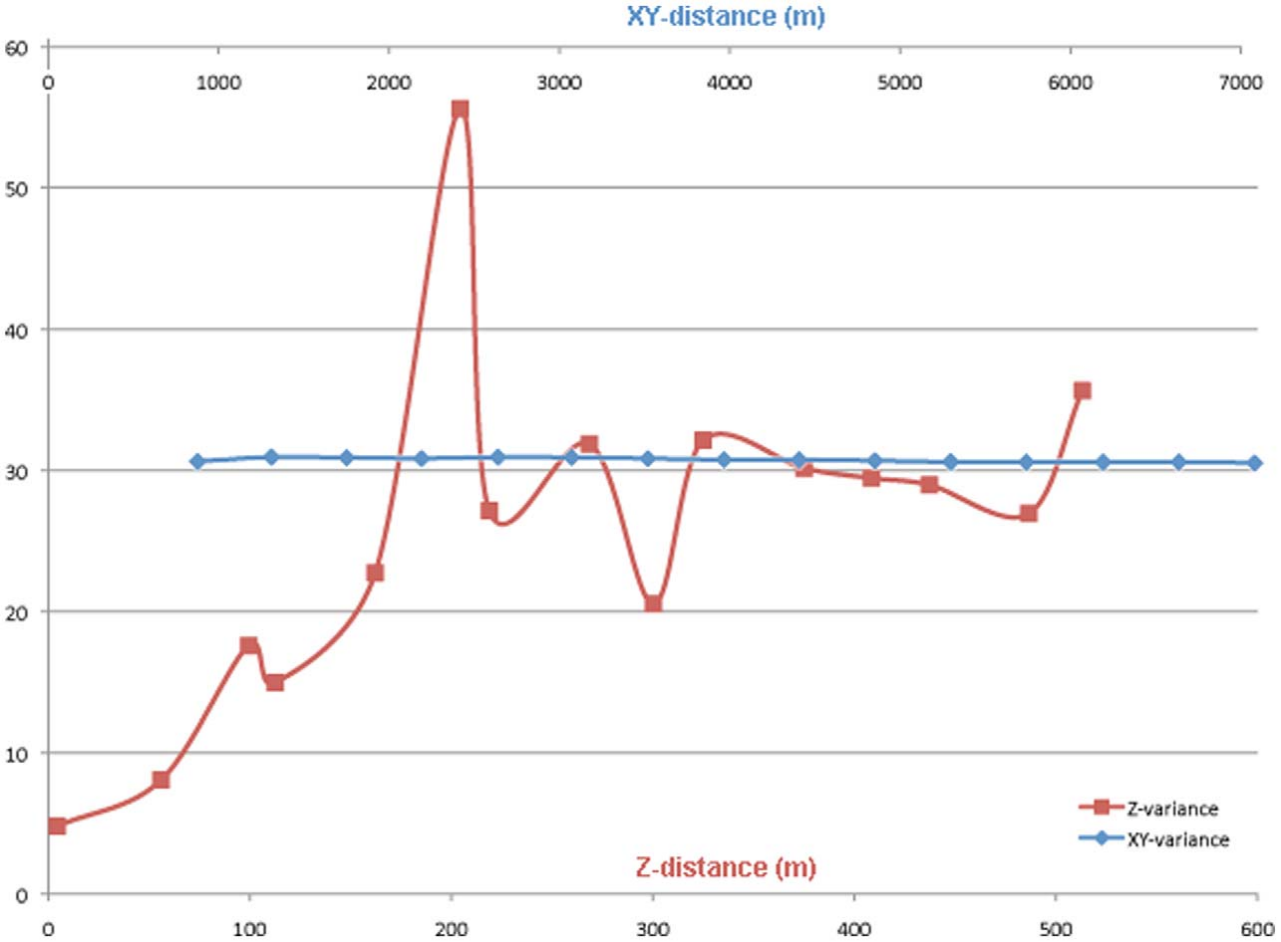
Semivariograms of acoustic backscatter with vertical (depth) and horizontal distances (x,y). Lag distance is on the x-axis with semivariance on the y-axis.

By examining the distribution of acoustic scatterers, we were able to determine ideal binning parameters and analysis approaches. The most apparent acoustic feature in the TOTO was a deep scattering layer with a mean width of approximately 200 meters centered at ca. 500 meters depth. Mean volumetric backscatter at 400–600 meters was greatest at the southern edge of our study site and on the western edge of the basin (Sv_mean_ = −71.03 dB) and weakest at eastern edge of the basin (Sv_mean_ = −76.3 dB). The acoustic density of scatterers was significantly dispersed in the horizontal (x–y) dimension throughout our study area (Moran's i = −0.02, p<0.01). This correlates with our qualitative observations that the primary scatters were distributed in layers rather than discrete patches. In contrast, the numbers and sizes of single targets per m^2^ were patchily distributed in the horizontal dimension (Moran's i = 0.07, 0.05 respectively, p<0.01). Semi-variograms for acoustic density in the x–y dimensions corroborated these findings with no discernable sill in the variance (Figure 2). For density as a function of depth (the z-axis), semivariograms showed a consistent sill at 200 m corresponding to the mean width of the deep scattering layer (Figure 2). All acoustic and oceanographic data were subsequently binned in 200-meter increments from 0 to 1000 meters depth and in 1 km horizontal distance.

**Figure 3:**
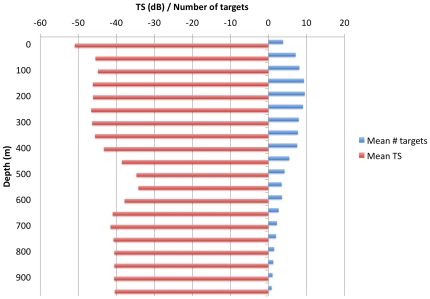
The mean number of targets, target size, and mean scattering volume with depth for the entire study area. There was a peak in 9 targets/2500 m^2^ at 200–250 m, a peak in target size of −35 dB at 550–600 m, and a peak in mean scattering volume at 500 m.

Patterns in the distribution of detected single targets were similar to overall scattering volume. Acoustically detected single targets ranged between 1–10 targets per 2500 m^2^ and had target strengths of −50 dB to −35 dB in size (Figure 3). The number of targets was greatest at 200 meters in depth with just below 10 targets per 2500 m^2^. The largest acoustic scatterers were between 500–600 meters in depth and had mean target strengths of −35 dB, and the depth of these larger scatterers often overlapped the bottom of the primary scattering layer. In contrast to overall backscattering density patterns, we found both the size and the number of single targets per m^2^ was not significantly different along the southern and western edge of the basin compared to the northern and eastern portion of the bay (Sv_mean_ = −40.7 and −40.8 dB; mean number of targets = 1.18 and 1.16 resepectively). However, without visual confirmation such as cameras or net tows, we are unable to differentiate among various scatterers including the various prey items available to beaked whales.

**Figure 4:**
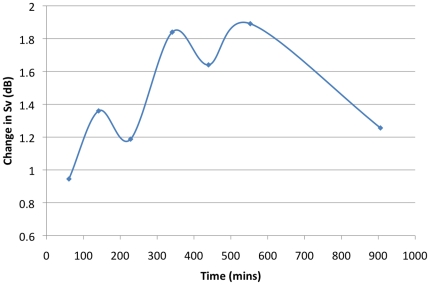
The difference in mean scattering volume at the center of each clover plotted against the time interval. The average change in backscatter was 1.9 dB over 550 minutes.

The temporal analysis at the center of each clover showed a maximum change in mean DSL scattering volume of 5.6 dB·m^−3^ over a period of 5.5 hours (Figure 4). By fitting a line through all of the clover center points, we calculated a mean change of 1 dB·m^−3^ over 3.2 hours suggesting a relatively static scattering layer over time. While we repeatedly observed a diel vertical migration at sunrise and sunset, the scattering volume at depth did not change significantly within or among days as indicated above. We also found no significant effect of time on whale relative foraging effort (t-test, mean of 53.3% of click duration during daylight, 18.1% sd) thus time of day was not included in our final analyses.

**Figure 5:**
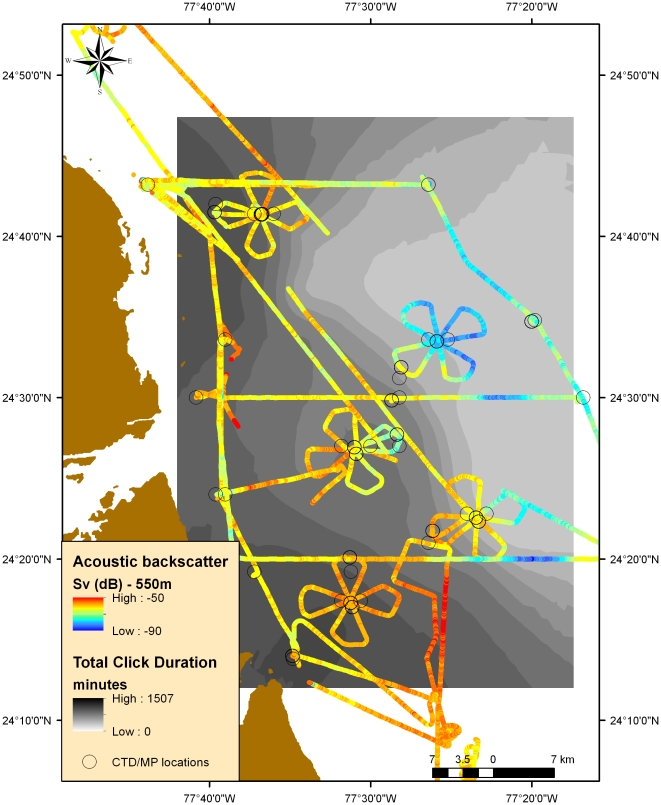
This plot shows interpolated relative foraging density at the extent of the hydrophone arrays in light to dark grey and backscatter at 550 meters from blue to red points representing low to high values. Circles are locations of CTD and MP casts. The western and southwestern areas of the study area had higher foraging effort and greater mean scattering volume.

There were a total of 1857 detected Blainville's beaked whale foraging events (click trains) with durations of up to 69 minutes per event. While it is extremely difficult to detect the number of animals involved in a click train, we used the total amount of time click trains were detected as a proxy for foraging effort. We found the western and southwestern hydrophones had the greatest durations of foraging clicks (Figure 5). There was significant spatial variability in foraging patterns as the total duration of clicks at each hydrophone varied from 0 minutes to over 1500 minutes for the duration of our cruise.

**Figure 6:**
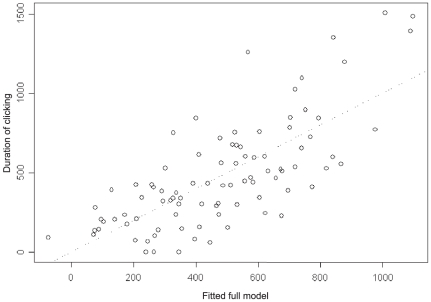
The full model 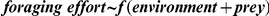 with response plotted against fitted variables.

GLM and GAMs were fit with relative foraging effort as the response against environmental and prey predictor variables. Relative foraging effort was normally distributed with constant variance. In addition, additive models did not offer increased explanatory power over linear models. Iteratively fit GLMs resulted in seven significant variables (Table 1). Bottom depth, salinity from 600–800 meters, and temperature from 200–400 meters had significant and negative effects on foraging effort while backscatter from 400–600 meters, number of single targets from 800–1000 meters, and salinity from 400–600 meters had significant and positive effects on foraging effort (Table 2). The full model explained 54% of the variance in foraging effort of beaked whales (Figure 6). The contrast between salinity at 400–600 meters and 600–800 meters suggests a halocline might have structured foraging effort. Reduced models including only prey variables explained only 34% of the variance in beaked whale foraging. The prey-only models resulted in positive relationships between foraging effort and volumetric backscatter between 400–600 m and single targets between 600–800 m but negative relationships with number of targets between 200–400 m and backscatter between 800–1000 m. Environment only models explained 43% of the variance and had positive relationships with surface salinity (0–200 m) and temperature (400–600 m) while the model showed a negative relationship between foraging effort, bottom depth, and temperature between 200–400 m. These models show good predictive power for foraging effort using environment or prey variables, but the model with the best explanatory power included both prey and environmental predictors.

**Table 1:** Final generalized linear model output and significance values.

	Parameter	Std. Error	t value	P value
(Intercept)	−158415	113978.75	−1.39	0.168
Backscatter (400–600 m)	4.1	0.82	5.01	0.000
Number of Targets (800–1000 m)	1620.5	529.04	3.06	0.003
Salinity (400–600 m)	18572	2925.46	6.35	0.000
Salinity (600–800 m)	−13399	4636.91	−2.89	0.005
Temperature (200–400 m)	−1862.5	375.36	−4.96	0.000
Bottom Depth	−1.3	0.22	−5.75	0.000

**Table 2:** Comparison of generalized linear model covariates for prey, oceanography, and prey-oceanography combined.

	Full	Prey	Env
**Parameters**	*Depth*	BS400	*Depth*
	BS400	*BS800*	*Temp200*
	NumT800	*NumT200*	Temp400
	Sal400	NumT800	Sal000
	*Sal600*		
	*Temp200*		
**AIC**	1193.495	1253.775	1224.106
**r^2^**	0.54	0.34	0.45

Negative relationships are shown in italics.

## Discussion

Research on oceanic top predators such as deep-diving beaked whales requires a suite of technologically advanced tools and analyses. The hydrophone range at AUTEC allowed an unprecedented measurement of the foraging habitat and behavior of these elusive predators. Distribution and density in the deep scattering layer were measured using fisheries acoustics allowing for high-resolution sampling of potential prey with depth. While species identification is near impossible with a single frequency echosounder particularly in diverse assemblages such as the deep scattering layers [41], we were able to measure relative density of a multispecies aggregation and single targets that include deep sea fish and squid, both primary prey species for Blainville's beaked whales [8], [42]. Physical oceanographic profiles were taken from CTD casts and microstructure turbulence profiles to examine depth-specific patterns in the pelagia that may serve to aggregate prey or assess suitable beaked whale foraging habitat. We were able to identify discrete foraging events in the TOTO and relate foraging effort to oceanography and prey by combining these advanced measurement techniques in a regression-modeling framework.

Deep scattering layers are common in the world's oceans and serve as an important forage base in often oligotrophic open ocean systems [43], [44]. We found a single DSL in the TOTO that ranged from 400–600 meters in depth, had a mean Sv (−73.2 dB·m^−3^), and showed minimal change over time (e.g. vertical migration). Diel vertical migration is a common feature of DSLs where organisms stay below the photic zone during the day to avoid predation and migrate to the surface at night to feed [44], [45], [46]. Static, non-migratory layers may be composed of different organisms than migratory layers or may be the same organisms at different life stages or facing different energetic requirements [18], [43], [47]. Although no trawl studies have been done examining the DSL in the TOTO, DSLs in the broader Atlantic have been identified as primarily myctophids and pelagic crustaceans by weight with occasional catches of large cephalopods [48], [49], [50]. Due to the high diversity of organisms within the DSL and difficulty in sampling at depth, identifying DSL distribution to species is difficult but relative density offers a valuable snapshot of potential beaked whale prey.

Blainville's beaked whales are known to feed primarily on cephalopods and secondarily on myctophids, two of the main components found in deep scattering layers [4], [51]. Analyzed dive records from tagged Beaked whales showed dives up to 1590 m [7] with foraging effort beginning at a mean depth of 400 m, continuing through the bottom of the dive and into the ascent, and ceasing clicking at a mean depth of 790 m [6], [8]. These depths agreed with our findings from generalized linear foraging models where both DSL density between 400–600 meters and single targets greater than 800 m were important in predicting foraging effort. Analyses of the returned echoes on acoustic tags from *Mesoplodon* also showed a higher capture rate at deeper depths and on larger targets (presumably squid) compared to smaller and shallower targets (presumably myctophids as part of the DSL [8], [42]. Additionally, including deep single targets improved our foraging models further supporting previous findings of beaked whale foraging behavior [6], [8].

Blainsville's beaked whales preferentially used the western edge of the TOTO for foraging and showed no time of day effect on foraging effort. The lack of a diel pattern in vertical distribution of backscatter was echoed by a lack of diel patterns in beaked whale click rates. As beaked whales often forage deep in the water column and below the photic zone [2], [8], it is not surprising that time of day had no significant effect (but see [31]). These results also agree with diel analysis of tag-derived dive behaviors where six Blainville's beaked whales showed similar foraging patterns between day and night, although there was greater time spent at the surface and fewer “bounce” dives at night [52], [53]. Bounce dives, where beaked whales perform a series of increasingly shallower dives after a single deep dive, were suggested as a method of predator avoidance during day rather than searching for prey.

Generalized linear models showed both environmental and prey predictor variables were important factors influencing Blainville's beaked whale foraging effort. Previous habitat models in the TOTO found that adult Blainville's beaked whales were found at shallower bottom depths (<1000 m) and at higher gradients in depth [3], [9]. We found a similar pattern with depth serving as an important predictor in both reduced and full models. Tag data from Hawaii suggested beaked whales use deeper waters (>1150 m) for foraging dives, but localized upwelling in the Bahamas may result in higher prey and in turn beaked whale sightings [9], [53]. Vertical water column structure also provided predictive value of beaked whale foraging effort particularly salinity and temperature between 200–600 meters. The gradient between salinity at 400 and 600 meters in the full model and temperature at 200 and 400 meters in the environment model may be caused by a pycnocline that is correlated with DSL distribution. Ferguson et al. [27] found the opposite trend with depth (<2000 meters) in the eastern tropical Pacific, but a similar trend for thermocline strength having a positive relationship with *Mesoplodon* sightings. While vertical profiles of oceanographic parameters are not always available for marine predator habitat models, cetacean habitat models in the California current were equally successful using remotely sensed surface variables as *in situ* measures [54], although the vertical structure in the TOTO may differ greatly from the upwelling-driven California current.

Including measures of environmental variables and prey variables explained more variation in beaked whale foraging than either model alone. When possible, both data sources should be included in cetacean foraging models but it is important to ensure both prey and predator measures are collected at consistent and appropriate spatial and temporal scales (e.g. [55]). Because the reduced model including only environmental variables had greater predictive power than the model with only prey variables, beaked whales were possibly using environmental features (e.g. bottom depth, temperature) to find their prey at the broader scales measured in this study. An alternative hypothesis is that acoustically similar scattering layers may differ in the amount of prey that is of interest to beaked whales requiring either a multi-frequency approach (e.g. [56]) or additional sampling gear necessary to tease apart the composition of the deep scattering layer. From analyses of fine-scale foraging behavior (e.g. individual foraging events from tag records) relative to environmental and prey correlates, predictive value from prey data might be equally if not more important than environmental variables as shown in models of species such as spinner dolphins (*Stenella longirostris*) in Hawaii [57], humpback whales (*Megaptera novaeangliae*) in the Gulf of Maine [23] and fin (*Balaenoptera physalus*), minke (*Balaenoptera acutorostrata*) and sei whales (*Balaenoptera borealis*) in Greenland [58]. Because animals interact with their environment at a variety of scales, explicit attention to spatial and temporal scales of ecological processes, sampling methods, and data processing are necessary when making habitat inferences.

Habitat models of both sightings and individual movement data have commonly used hydrographic, bathymetric, and less commonly prey variables to predict the distribution and behavior of marine top predators across a number of spatial and temporal scales. For four dolphin species in the eastern tropical Pacific, thermocline strength, thermocline depth, and surface chlorophyll were all important in predicting habitat [59]. Dolphin sightings were analyzed at a range of scales (2–120 km) relative to oceanographic features and no scale dependence was found [59] suggesting that dolphins were most likely using their oceanographic environment at broader scales than were measured. Torres et al. [55] found prey items to be uninformative for foraging bottlenose dolphins in the Florida keys, but used intermittent trawls to describe the prey community potentially leading to a scale mismatch between sampling of prey and predator foraging behavior. While we included diffusivity in our models, it was calculated using measured turbulence levels in the water column often varying at much finer scales than 1 km horizontally or 200 m vertical. It was not surprising that diffusivity did not have a significant effect on beaked whale foraging, but finer scale analyses between acoustically detected organisms and diffusivity may better explain the physical and biological structure in the DSL. Our results suggest beaked whales may use multiple spatial scales to forage on various prey types, potentially tracking broad scale features and using echolocation to find the local maxima in prey density.

Understanding the habitat of marine mammals is important for predicting their distribution, particularly with respect to anthropogenic impacts. Generalized additive models were used to identify suitable sites for harbor porpoise (*Phocoena phocoena*) marine protected areas as a function of bathymetric and hydrographic variables in Scotland highlighting the utility of habitat models in marine conservation [25]. Bottlenose dolphin (*Tursiops truncatus*) habitat use within the Moray Firth in Scotland was analyzed to understand how marine mammal species use and existing marine reserve at multiple spatial scales [60]. These studies illustrate how habitat modeling can be a powerful tool that can inform spatially adaptive management of pelagic predators. In the TOTO, our findings suggest increased use of the western edge of the basin compared to the east, which may correspond to an increased risk from sonar exercises.

Little is known about the distribution much less the ecology of beaked whales due to long, deep dives, and short surface intervals [3], [6]. Most of the diet and life history data originate from stranded animals leaving a gap in our knowledge of their foraging behavior [9]. Our study provides an initial analysis of Blainville's beaked whale foraging habitat through the use of advanced acoustic technology and modeling. Even though we focus on a limited area and time snapshot, we found both prey metrics (number of single targets and density of the DSL) and environmental features (salinity, temperature, bottom depth) influenced foraging effort. While further work is necessary to determine whether this foraging habitat model could be extrapolated to other seasons or areas, it provides necessary insight into how beaked whales interact with their environment. Ultimately, both prey distribution and environmental features could inform spatial management approaches for these elusive and deep diving species in the Bahamas.
